# Impact of *Plasmodium falciparum* infection on DNA methylation of circulating immune cells

**DOI:** 10.3389/fgene.2023.1197933

**Published:** 2023-07-04

**Authors:** Dareen Almojil, Aïssatou Diawara, Issiaka Soulama, Mame Massar Dieng, Vinu Manikandan, Samuel S. Sermé, Salif Sombié, Amidou Diarra, Aissata Barry, Sam Aboubacar Coulibaly, Sodiomon B. Sirima, Youssef Idaghdour

**Affiliations:** ^1^ Program in Biology, Division of Science and Mathematics, New York University Abu Dhabi, Abu Dhabi, United Arab Emirates; ^2^ Centre National de Recherche et de Formation sur le Paludisme, Ouagadougou, Burkina Faso

**Keywords:** malaria, *Plasmodium falciparum*, DNA methyaltion, methylome, host parasite interactions, TNF

## Abstract

The regulation of immune cell responses to infection is a complex process that involves various molecular mechanisms, including epigenetic regulation. DNA methylation has been shown to play central roles in regulating gene expression and modulating cell response during infection. However, the nature and extent to which DNA methylation is involved in the host immune response in human malaria remains largely unknown. Here, we present a longitudinal study investigating the temporal dynamics of genome-wide *in vivo* DNA methylation profiles using 189 MethylationEPIC 850 K profiles from 66 children in Burkina Faso, West Africa, sampled three times: before infection, during symptomatic parasitemia, and after malaria treatment. The results revealed major changes in the DNA methylation profiles of children in response to both *Plasmodium falciparum* infection and malaria treatment, with widespread hypomethylation of CpGs upon infection (82% of 6.8 K differentially methylated regions). We document a remarkable reversal of CpG methylation profiles upon treatment to pre-infection states. These changes implicate divergence in core immune processes, including the regulation of lymphocyte, neutrophil, and myeloid leukocyte function. Integrative DNA methylation-mRNA analysis of a top differentially methylated region overlapping the pro-inflammatory gene TNF implicates DNA methylation of TNF *cis* regulatory elements in the molecular mechanisms of TNF regulation in human malaria. Our results highlight a central role of epigenetic regulation in mounting the host immune response to *P. falciparum* infection and in response to malaria treatment.

## 1 Introduction

Despite the long history of malaria (Laveran, 1881; Poinar, 2005; Cox, 2010), the disease remains a major public health concern with an estimate of 619,000 deaths globally in 2021, 78.9% of which are children under the age of five in Sub-Saharan Africa (WHO, 2022). Human malaria is attributed to five species of *Plasmodium*, among which *P. falciparum* and *P. vivax* are the most studied due to their high prevalence in causing the disease (Perkins, 2014; Sato, 2021). Malaria has been challenging to eradicate due to the multi-layered host and parasite factors, their interaction with each other and with the environment (Idaghdour et al., 2012; Dieng et al., 2020). Host-parasite interactions often occur over relatively short evolutionary time scales, and it has been suggested that epigenetic and transcriptional mechanisms play important roles in these interactions in malaria (Gómez-Díaz et al., 2012; Idaghdour et al., 2012; Cheeseman and Weitzman, 2015). Understanding the nature and role of these molecular mechanisms in the context of how host immune cells respond to *Plasmodium* infection and to treatment with potential consequences on the course and outcome of infection is important to our overall understanding of how host–parasite interactions impact the pathology of the disease.

The role of epigenetic processes in complex diseases is becoming increasingly evident (Khyzha et al., 2017; Deng et al., 2019; Wang et al., 2019; Dobbs et al., 2022; Bouyahya et al., 2022; Ding et al., 2022; Kaimala et al., 2022). Epigenetic studies have begun to establish mechanistic links between epigenetic regulation and diseases susceptibility such as for rheumatoid arthritis (Liu et al., 2013) and type I diabetes (Rakyan et al., 2011) and also variation in immune-related gene expression traits (Lim et al., 2013; Obata et al., 2015), immune cell differentiation processes (Lai et al., 2013; Netea et al., 2015; Sellars et al., 2015; Rusek et al., 2018; Sun and Barreiro, 2020), immune memory genesis. For instance, Zhang and colleagues investigated the role of epigenetic mechanisms and DNA methylation in immune cell lineage differentiation and memory generation and reported specific DNA methylation patterns involved in the differentiation of memory cell subtypes with shared epigenetic regulation mechanisms across different immune cell lineages (Zhang et al., 2022). Despite the growing appreciation of the role of host epigenetics in the host immune response to pathogens such as *Mycobacterium tuberculosis* (Pennini et al., 2006; Gómez-Díaz et al., 2012; Cheeseman and Weitzman, 2015; Pacis et al., 2015), *Leishmania donovani* (Marr et al., 2014), and *Toxoplasma gondii* (Sabou et al., 2020), knowledge on the role of host epigenetic modifications in malaria is limited. Most malaria investigations published to date focused on the epigenetics of the parasite *P. falciparum* (Batugedara et al., 2017; Cortés and Deitsch, 2017; Gupta et al., 2017; Arama et al., 2018; Schrum et al., 2018). Investigations of host epigenetics in malaria were largely limited to candidate gene approaches including genes mediating host resistance/susceptibility (Stern et al., 2016; Lessard et al., 2017; Nisar et al., 2022), innate and adaptive immunity-related genes (Dobbs et al., 2020; Loiseau et al., 2020; Dobbs et al., 2022) and immune cell differentiation processes (Soon and Haque, 2018; Studniberg et al., 2022). Other human malaria studies have focused on the role of miRNAs in response to malaria (El-Assaad et al., 2011; Dkhil et al., 2017; Dieng et al., 2020; Gupta and Wassmer, 2021). We currently lack a comprehensive understanding of how *P. falciparum* infection impacts the host methylome during uncomplicated blood stage malaria.

In this study, we address this gap in knowledge by performing a longitudinal study to document and investigate the nature of genome-wide DNA methylation changes taking place *in vivo* in circulating immune cells of African children upon *P. falciparum* infection and treatment. We take advantage of a robust matched study design where the same group of children are sampled prior to infection, during symptomatic *P. falciparum* infection and 3 weeks after treatment. The results provided an unbiased view of the dynamics of methylation changes during the course of malaria infection and after treatment, identified the genomic regions impacted by these changes and highlighted epigenetic regulation as a major modulator of circulating immune cell function in human malaria.

## 2 Materials and methods

### 2.1 Study design and population

**FIGURE 1:**
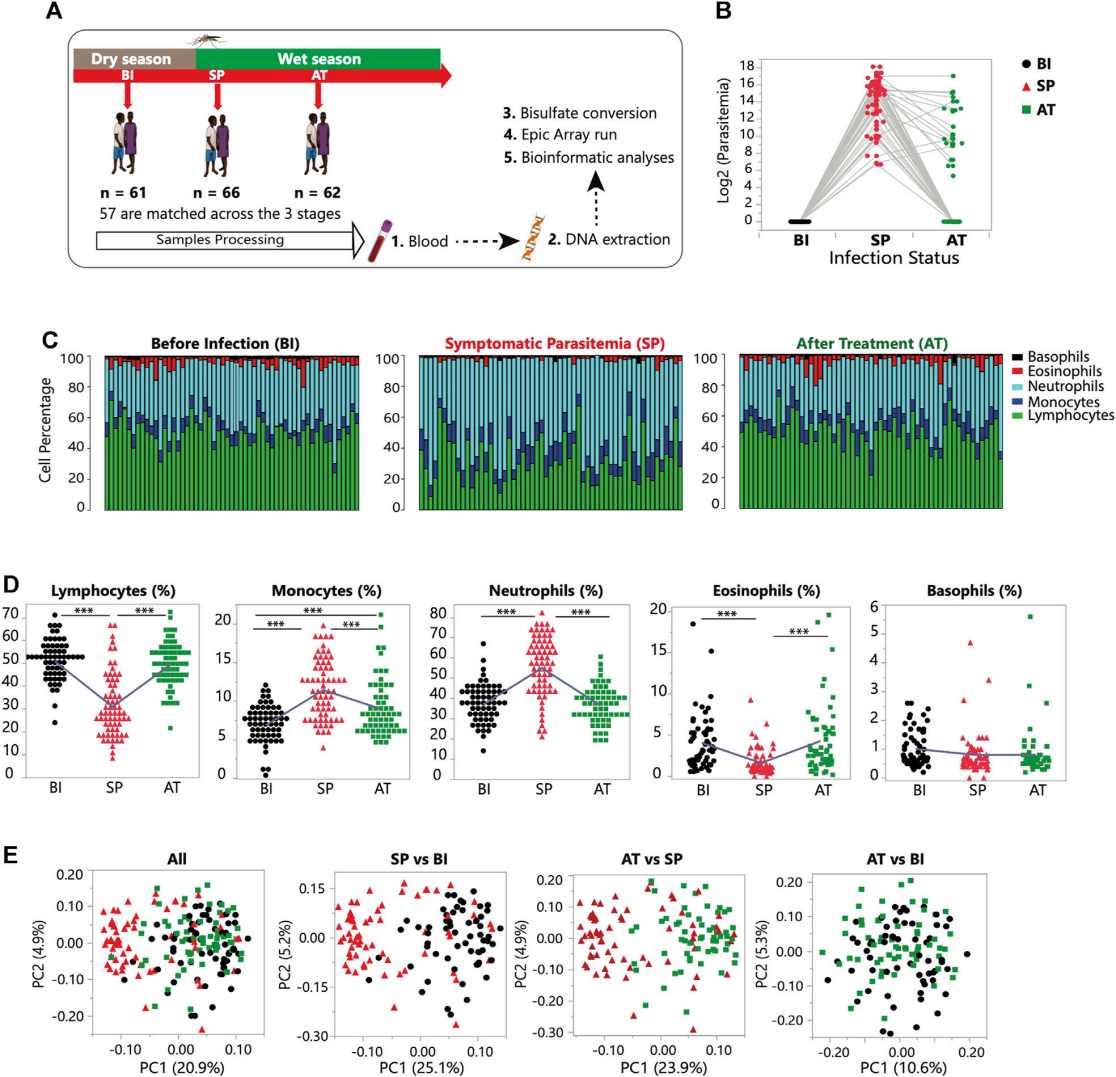
Study design and longitudinal dynamics of genome-wide DNA methylation profiles in malaria. **(A)** Study experimental design. The children were longitudinally sampled at three stages: Before Infection (BI, *n* = 61); Symptomatic Parasitemia (SP, *n* = 66) and After Treatment (AT, *n* = 62). **(B)** Parasitemia levels (log2) in the three stages profiled: Before Infection (*n* = 61, in black); Symptomatic Parasitemia (*n* = 66, in red) and After Treatment (*n* = 62, in green). The grey lines connect the matched samples. **(C)** Changes in the proportion of five white blood cell types during the course of the three stages sampled. Each bar represents one individual and the proportion of each cell type is shown for each stage. White blood cell count data was generated using the hematology analyzer Pentra C200 (HORIBA). **(D)** The percentage (%) of white blood cells (WBCs), including of lymphocytes, monocytes, neutrophils, eosinophils, and basophils, is plotted for each study participant in three groups: BI (*n* = 61), SP (*n* = 66), and AT (*n* = 62). Statistical significance for the mean difference between all pairs was tested using Tukey-Kramer HSD. The sign (***) indicates a *p*-value of <0.001. **(E)** Principal component analysis (PCA) of the DNA methylome for the three stages combined and for each pairwise comparison (SP vs. BI; SP vs. AT and AT vs. BI). Color and shape of the data point is referring to its infection status; BI = green square, SP = red triangle, and AT = black circle.

The study protocol and informed consent procedures were approved by the Institutional Review Board of New York University Abu Dhabi (UAE, protocol number 011–2015) and the Comité d’Ethique pour la Recherche en Santé au Burkina Faso (Ministry of Health, Burkina Faso; protocol number 2015-02-018). Assent for children older than 4 years of age and informed consent from parents or legal guardians of all study participants were obtained following the approved study protocol. Inclusion criteria are *(i)* residence in Bounouna and Nafona villages in the malaria-endemic Banfora district located 441 km from Ouagadougou the capital of Burkina Faso, West Africa, *(ii)* age between two and ten, *(iii)* no disease symptoms or chronic disease history (including sickle cell disease) based on medical records and physical assessment by a doctor at the CNRFP clinic in Banfora and *(iv)* no parasitemia at enrolment during the dry season (based on thick blood smear analysis and subsequently confirmed using RNASeq data). Transmission of malaria in Burkina Faso is markedly seasonal with a peak during the rainy season where around 60% of the total annual number of malaria episodes occurs (Tiono et al., 2013). Following enrollment, the study participants were followed on a weekly basis for up to 6 months. Phenotyping and sampling were done at three time points: Before Infection (BI), at the beginning of Symptomatic Parasitemia (SP), and 3 weeks After artemether/lumefantrine (Coartem) Treatment (AT) Figure 1A. In total, 150 children were recruited of which approximately 60 were carefully selected for the methylation study based on completeness of the data (Supplementary Data).

### 2.2 Blood samples and data collection

Blood samples (∼10 ml) and patient data were collected at the CNRFP clinic in Banfora. To minimize heterogeneity due to the circadian rhythm and technical reasons, blood samples were collected between 9:00 a.m. and 2:00 p.m. and processed immediately after collection following the same protocols throughout the various downstream experiments. One ml of blood was collected for DNA isolation. The remaining blood was used for standard thick blood smear analysis and to generate hemoglobin level and total cell count data including lymphocytes, neutrophils, monocytes, eosinophils and basophils. White blood cell count data was generated using the hematology analyzer Pentra C200 (HORIBA). Hemoglobin and G6PD genotypes were determined as previously described (Bougouma et al., 2012). Parasitaemia was calculated using manual counting of Giemsa-stained thin and thick blood smears and bright-field microscopy. Other covariates of interest such as age, sex and location were collected from all participants. Participant demography, biochemical and cell count data are summarized in Supplementary Data and Supplementary Table S1.

### 2.3 DNA isolation and methylation profiling

Genomic DNA was extracted from whole blood using Qiagen DNAeasy Blood and Tissue Kit (Qiagen) following the manufacturer’s instructions. DNA quality and concentration were checked using nanodrop and qubit instruments, respectively. A total of 50 ng of genomic DNA was used for genome-wide DNA methylation profiling at the Australian Research Genomics Facilities (ARGF, Melbourne) using Illumina’s Infinium MethylationEPIC (HM850 K) platform. Briefly, genomic DNA was bisulfite-converted using the EZ DNA Methylation kit (Zymo) then assayed by the Illumina Infinium MethylationEPIC BeadChip array that interrogates over 850,000 CpG methylation sites. Quality of the bead chip array was assayed by sample-independent and dependent controls for staining, extension, target removal, hybridization, bisulfite conversion, allele-specific extension and primer extension.

### 2.4 Data processing and quality control

Raw methylation intensities were processed in R (version 3.5.1) using the R/Bioconductor package *minfi* (Aryee et al., 2014). For each sample, the estimated methylation level at each CpG site is expressed as a beta value (*β*), which is the ratio of the methylated probe intensity to the overall intensity [i.e., calculated using the following formula C/(C + T +100), where C and T are the intensities of methylated and unmethylated signals, respectively (Pidsley et al., 2016)]. The produced *β* values were then adjusted using the 500-cell type-informative probes reported by Houseman et al. (2012) to account for differences in methylation arising from differences in cell type composition in whole-blood samples. *β* values were adjusted by using penalized regression to regress out the cell types using the function “adjust.beta” following the Houseman et al. (2012) procedure. The new adjusted *β* values were then logit transformed to the adjusted M values that were subsequently used for the statistical analyses. WBC composition data measured in the lab was also directly used as covariates to account for cell composition and the results were similar to those of the Houseman et al. (2012) method.

To reduce the non-biological differences between probes and to adjust for the different probe design types present in the 850 K architecture, the intensities of the signal were corrected for background fluorescence and technical variations using functional “*funnorm*” normalization implemented in *minfi* for both adjusted *β* and adjusted M values (Supplementary Figure S1). Then, stringent filtering was applied to filter out probes based on the following criteria: *(i)* probes overlapping single nucleotide polymorphism (SNP) with minor allele frequency (MAF) > 0.05 in 1 K Genome data (using the “*dropLociWithSnps”* function), *(ii)* probes on X and Y chromosomes, and *(iii)* cross-reactive probes as recommended and listed by Pidsley et al. (2016). This filtering resulted in a total of 794,698 CpG sites retained for downstream analyses.

### 2.5 DNA methylation data analysis

Methylation distribution data analysis and principal component analysis (PCA) were done on the full 794,698 CpG loci dataset using JMP Genomics (SAS Institute). Longitudinal changes in DNA methylation for the 794,698 CpG loci were assessed using DMRcate (Peters et al., 2015) for each infection status contrast: Before Infection vs. Symptomatic Parasitemia, Symptomatic Parasitemia vs. After Treatment, and After Treatment vs. Before Infection using the following linear mixed effect model; (*∼Infection_Status + Age + Sex + Individual*) (Supplementary Data). Age and sex effects are expected to be marginal given the matched study design nonetheless they were included in the model as covariates. Given the longitudinal nature of the data (i.e., repeated measures), the “Individual” effect was accounted for by including it in the analysis as a blocked parameter. The run settings for DMRcate were adjusted based on Mallik et al. (2019) which showed that power and precision of the DMRcate run were best achieved when lambda was set to 500, and parameter C to 5. The *p*-value cutoff measure for the identification of differentially methylated probes and regions was set to 0.001. Results were then corrected for multiple testing using the Benjamini–Hochberg method. The annotations of the methylation probes used in the analyses were obtained from the R/Bioconductor package IlluminaHumanMethylationEPICanno.ilm10b4. hg19 (package version 3.7). Visualizations of the temporal and spatial changes in DMPs and DMRs were plotted using the ggplot2 R package (version 3.4.0). DNA methylation age was estimated using the epigenetic clock model developed by Horvath et al. (2018) and implemented in the methyAge package in R 4.2.2 (R Core Team). The model uses 391 CpGs measured from six different tissues including whole blood from participants of a wide age range 0–94 years (Horvath et al., 2018). The model was chosen since our data was generated from children (median 4 years old; range; 2–9.1 years old) and the model has demonstrated better performance with children data (Wu et al., 2019; Kling et al., 2020; McEwen et al., 2020; Monasso et al., 2021). The model generated methylation age and acceleration values that were compared between the three infection stages investigated using ANOVA and Tukey’s HSD test.

### 2.6 Gene set enrichment analysis

We identified gene ontology (GO) cellular component, molecular functions and biological processes pathways that were significantly enriched for DMRs at cut-off *p*-value <0.01 using the gometh function of the missMethyl R package (version 1.10.0 gometh function) (Phipson et al., 2016). The gometh function included an adjustment for the underlying distribution of probes on the array to remove bias due to unbalanced distribution of probes per gene interrogated in the array. Cluego (Lee et al., 2005), available through the program Cytoscape (version 3.71) (Shannon et al., 2003), was then used to quantify enrichment scores and significance.

### 2.7 Correlation between TNF expression and methylation level

The expression levels of TNF (IQR normalized) of 40 children sampled at three time points, Before Infection, during Symptomatic Parasitemia and After Treatment were obtained from an RNA-seq dataset (Dieng et al., 2020; Abdrabou et al., 2021) generated from the same blood samples as the methylation dataset. This matched dataset provided an opportunity to assess the association between methylation and expression level of TNF. Normalized TNF levels of 40 children were correlated (Pearson method) with their matched adjusted beta values of 13 CpGs averaged located on the DMR that overlaps with the TNF gene locus. Expression-methylation correlations were tested separately for each of the three infection stage groups.

## 3 Results

Over a 3-month period, we recruited and sampled longitudinally a pediatric cohort of children in Banfora district in Burkina Faso. Phenotypic, clinical data and blood specimens were collected at three time points: Before Infection (BI), during Symptomatic Parasitemia (SP) and 3 weeks After Treatment (AT) Figures 1A, B. White blood cells (WBCs) are a central component of the immune system and their composition and response to the infection can modulate the course of the disease. Proportions of the five major WBCs (lymphocytes, monocytes, neutrophils, basophils, and eosinophils) were analyzed to assess the nature of changes taking place in response to *P. falciparum* infection and treatment (Figures 1C, D). Proportions of each cell type were compared between the three stages (Figure 1C). As the children transition to the SP stage, significant differences in the counts of monocytes, lymphocytes, neutrophils and eosinophils (*p* < 0.001) were observed. In particular, we highlight the significant depletion of the proportion of lymphocytes during the SP stage, and the increase in the proportions of monocytes and neutrophils (Figures 1C, D; Supplementary Table S2). In response to treatment, significant changes in cell proportions are observed again but in the opposite direction to the response to the infection (Figure 1D). Comparing the Before Infection (BI) and After Treatment (AT) groups shows that in general after treatment the children recover their pre-infection WBCs proportions with no significant differences observed.

### 3.1 Longitudinal dynamics of methylation profiles throughout the course of infection

To infer the correlation structure in the data, we performed principal component analysis (PCA) using the entire DNA methylation dataset that consists of normalized 794,698 CpG sites methylation levels from 189 samples representing the three infection stages (BI, SP and AT) (Supplementary Figure S1, S2). The first methylation principal component (PC1), indicating the variation and degree of differentiation among the three infection stages, explains ∼20% of the total variance and clearly captures the effect of *P. falciparum* infection on host DNA methylation profiles (Figure 1E; Supplementary Figure S2). PCA of pair groups captured the gradual temporal changes of *P. falciparum* infection (Figure 1E). Variance component analysis shows that infection status accounts for 41.8% of the total variance in PC1.

#### 3.1.1 Differentially methylated positions (DMPs)

**FIGURE 2:**
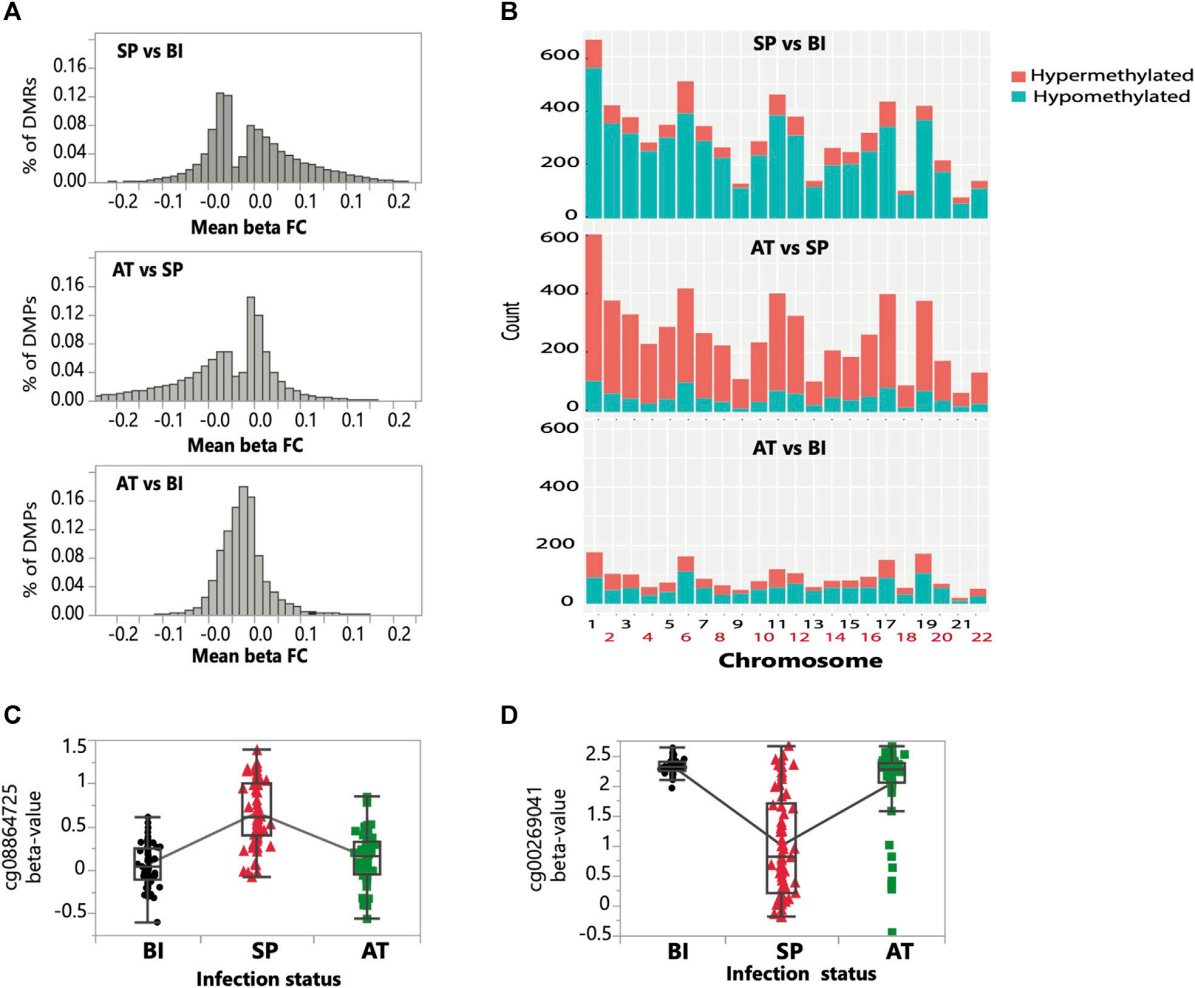
Genome-wide distribution of differentially methylated positions and regions throughout the three infection stages. **(A)** Distribution of hypermethylated (greater than zero) and hypomethylated (lower than zero) differentially methylated positions (DMPs) for the three pairwise comparisons (SP vs. BI; SP vs. AT, and BI vs. AT). **(B)** Chromosomal distribution of the counts of hypermethylated and hypomethylated differentially methylated regions (DMRs) for the three pairwise comparisons (BI vs. SP, SP vs. AT, and BI vs. AT). The bar height reflects the density of DMRs for each chromosome. The blue and red colors on the bars represent the counts of hypo- and hyper-methylated DMRs within the chromosome, respectively. **(C, D)** Examples of temporal changes in methylation of two randomly selected CpGs of opposite trend. **(C)** a CpG which has an increase in methylation in SP compared to BI and AT stages, and **(D)** a CpG which has an decrease in methylation in SP compared to BI and AT stages.

To evaluate the genome-wide DNA methylation differences and establish the direction of the changes in whole blood DNA during the course of *P. falciparum* infection, we performed differential methylation analyses on the 794,698 CpG sites in 189 samples. We used a mixed regression model that accounts for age, sex, and the individual effect (see Methods for details) to identify differentially methylated positions (DMPs) and regions (DMRs). We identified ∼79.9 K (10%), ∼73.2 K (9%) and 9 K (1%) DMPs passing the experiment-wide significance threshold (FDR <0.01) between the contrasts Symptomatic Parasitemia (SP) vs Before Infection (BI), After treatment (AT) vs Symptomatic Parasitemia (SP) and After Treatment (AT) vs Before Infection (BI), respectively, (Table 1). The temporal dynamics of DNA methylation profiles are shown in Figure 2. We note that 18 children did not clear the parasites from their blood after treatment. Differential methylation analysis between this group and the children who cleared the parasite revealed no differentially methylated CpGs (FDR <0.01) and only 11 CpGs at FDR <0.05 (Supplementary Figure S3).

**TABLE 1:** Number of differentially methylated positions (DMPs) and regions (DMRs) for both adjusted beta and M values obtained using *DMR*cate.

	Adjusted beta values (β)		Adjusted M values	
Contrast	DMPs	DMRs	DMPs	DMRs
BI vs. SP	80,127	6,845	79,971	6,818
SP vs. AT	73,321	5,789	73,210	5,767
BI vs. AT	9,130	2,015	9,040	2,005

Approximately three-quarters (59.2 K) of the total identified DMPs (i.e., ∼79.9 K) were shared by two pairwise comparisons (Symptomatic Parasitemia vs. Before Infection and After treatment vs. Symptomatic Parasitemia) and less than 1% (El-Assaad et al., 2011) were shared by the three groups (Supplementary Figure S4A). Through examining the methylation status and trend of DMPs (hypo vs. hyper methylation) between pairwise comparisons (Symptomatic Parasitemia vs. Before Infection and After Treatment vs. Symptomatic Parasitemia), we observed a switch from an overly hypomethylation state between the Symptomatic Parasitemia vs. Before Infection pairwise comparison, to an overly hypermethylation state between the After Treatment vs. Symptomatic Parasitemia pairwise comparison (Figure 2A). This was additionally highlighted by the Venn diagram which revealed significant overlaps of DMPs shifting from hypo to hyper-methylated states when comparing the Symptomatic Parasitemia vs. Before Infection and After Treatment vs. Symptomatic Parasitemia pairwise groups (Supplementary Figure S4B, C). This finding clearly shows reversal of the direction of methylation change following malaria treatments. This is consistent with the observation that the overall methylation state of children before infection and after treatment is similar (Figure 1E; Figure 2A). The number and magnitude of changes observed between states (Before Infection to Symptomatic Parasitemia and Symptomatic Parasitemia to After Treatment) were similar and largely correspond of reversal of methylation states which resulted in limited differences between After Treatment and Before Infection states (Figure 2A). Examples of these patterns of methylation reversal are shown for two CpG sites in Figures 2C, D.

#### 3.1.2 Differentially methylated regions (DMRs)

Expending the differential methylation analysis from CpG sites to DMRs, we identified ∼6.8K, ∼5.7K, and ∼ 2 K DMRs passing the experiment-wide significance threshold (FDR <0.01) between Symptomatic Parasitemia (SP) vs. Before Infection (BI), After Treatment (AT) vs. SP and AT vs. BI, respectively (Table 1). The temporal dynamic trend of methylation profiles of DMRs shows a similar pattern to that of DMPs (Figure 2B). While around 82% (5.6 K) of DMRs during infection (from Before Infection to Symptomatic Parasitemia state) were hypomethylated, we observed approximately 80% (4.6 K) of DMRs to be hypermethylated after treatment (from Symptomatic Parasitemia to After Treatment states). This finding confirms the inferred restoration of the pre-infection DNA methylation states. This was further confirmed when After Treatment and Before Infection methylation states were compared and showed low levels of methylation changes between the two states (Figure 2B). Plotting the density of DMRs across chromosomes for each of the three pairwise comparisons (SP vs. BI, AT vs. SP and AT vs. BI) while accounting for the CpG probe content in the EPIC array showed that the distribution of DMRs across chromosomes is not random with most changes taking place in chromosomes 1, 6, 11, 17, and 19 (Supplementary Figure S5). We highlight in particular the case of chromosome 6 that harbors the HLA region being one of the most impacted by methylation changes in our dataset. Collectively, our DMPs and DMRs analyses show that *P. falciparum* infection and malaria treatment have a significant impact on the DNA methylation states of children. Particularly noteworthy is the finding that the majority of global methylation changes occurring during the acute phase of the disease (Symptomatic Parasitemia) revert back to their pre-infection (Before Infection) states after treatment.

Next, to assess the effect of *Plasmodium* infection and malaria treatment on epigenetic age, DNA methylation age was calculated using the estimator developed by Horvath et al. (2018). The model performed well with correlations between DNA methylation age and chronological age >0.8 (*p* < 0.0001) in all three stages of the infection (Supplementary Figure S6A). The results show no statistically significant differences in group’s epigenetic age and acceleration means (Supplementary Figures S6B, C).

### 3.2 Gene set enrichment analysis

**FIGURE 3:**
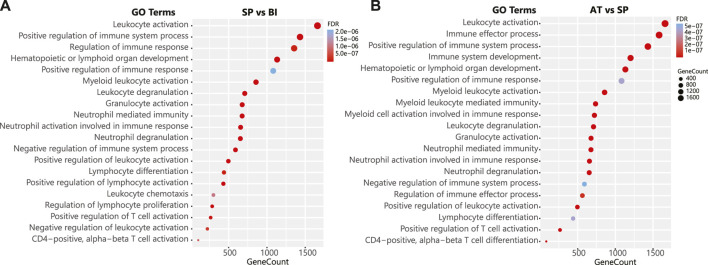
Pathway enrichment of differential immunological responses to malaria infection. **(A)** The top 20 statistically significant (FDR <0.01) pathways enriched for the contrast Symptomatic Parasitemia (SP) vs. Before Infection (BI). **(B)** The top 20 statistically significant (FDR <0.01) pathways enriched for the contrast After treatment (AT) vs. Symptomatic Parasitemia (SP). The *y*-axis indicates the pathway name, *x*-axis and the bubble size indicate the number of genes in each pathway. The color bar indicates the corrected FDR *p*-value, blue represents higher values (less significant), red represents lower values (more significant). Pathways are ranked based on their gene count. Pathway enrichment analysis was done using the gometh function of the missMethyl R package (version 1.10.0 gometh function). Grouping pathways to a non-redundant list was performed using ClueGO. For the full list of the statistically significant pathways see Supplementary Tables S1H, I.

Gene set enrichment analysis of differentially methylated regions revealed the impact of the methylation changes observed on immunological pathways in particular immunoglobulin production, leukocytes activation, myeloid leukocyte activation, neutrophil degranulation and T cell activation (Figure 3). Interestingly, the same pathways impacted upon infection were impacted in response to treatment which aligns with the DNA methylation reversal trend observed in our quantitative temporal dynamic analysis of DMPs and DMRs. Collectively, differential methylation and enrichment analyses show that global methylation patterns that are disrupted in response to *P. falciparum* infection are restored quantitatively and qualitatively leading to the restoration of the pre-infection functional and immunological states following malaria treatment.

### 3.3 Correlation between TNF expression and methylation levels of associated CpGs

**FIGURE 4:**
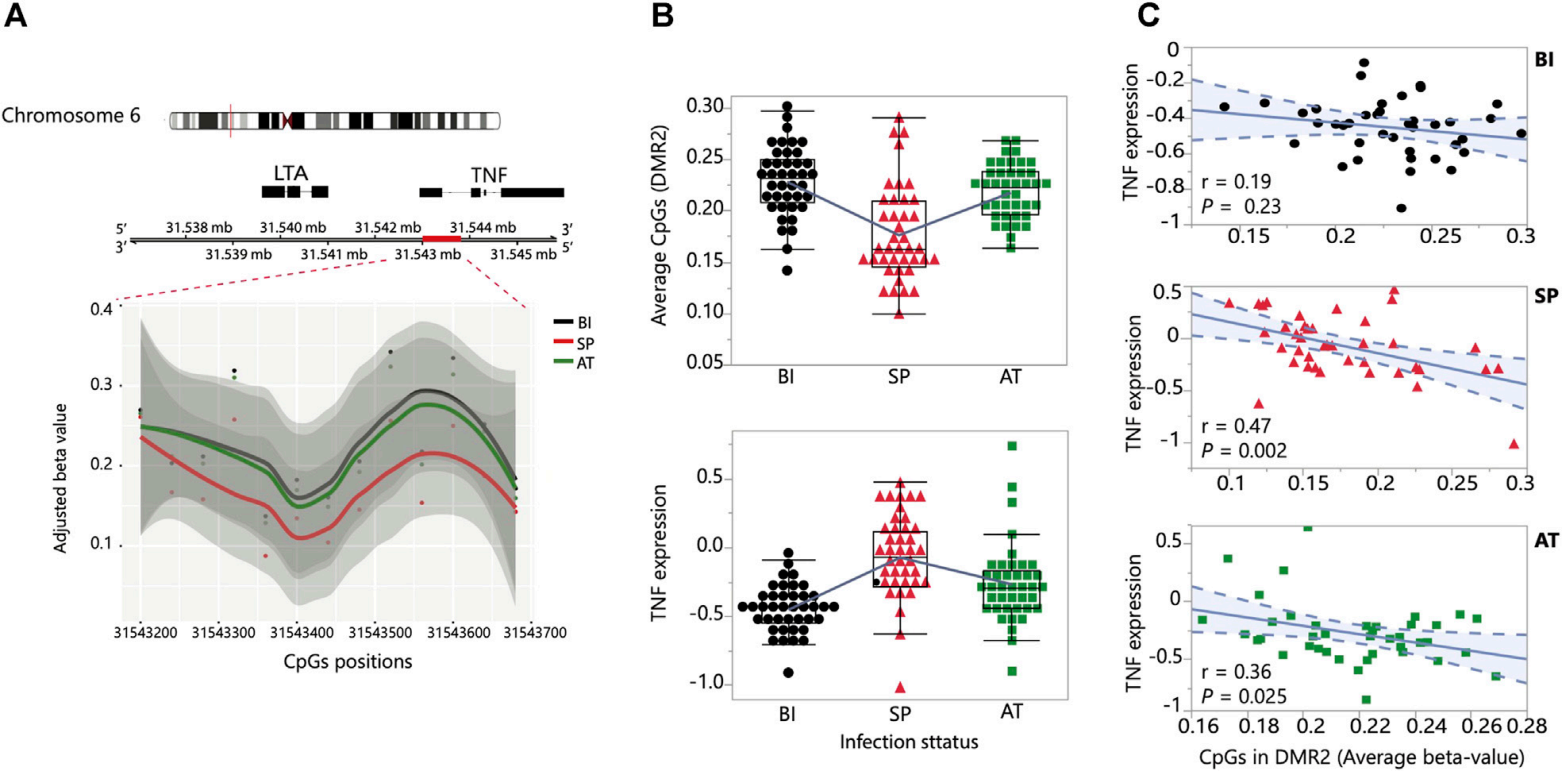
DNA methylation association with TNF expression. **(A)** A TNF-associated DMR (red bar) on chromosome 6 showing the temporal change in methylation state. The *y*-axis and *x*-axis show the adjusted beta-values and the positions of the CpGs in the DMR, respectively. **(B)** Temporal changes in methylation (top) and TNF expression (bottom) of the 40 children sampled at the three stages: Before Infection, Symptomatic Parasitemia and After Treatment (BI, SP and AT). **(C)** Correlation plots of the association between the CpGs adjusted beta values and level of TNF expression at the three stages: Before Infection (BI, black), Symptomatic Parasitemia (SP, red) and After Treatment (AT, green).

To gain insights into the effects of global methylation on gene expression regulation during *P. falciparum* malaria, we performed a focused analysis of the top most significant perturbation observed on chromosome 6 which implicates the TNF locus (Figure 4A). Allelic variation in this locus and closeby genes have been associated with severe outcomes following *Plasmodium* infection in multiple populations (Kwiatkowski, 1990; McGuire et al., 1994; Clark Taane et al., 2009). The DMR identified in our analysis is located right upstream of the TNF locus and contains 13 CpGs (Figure 4A). The 13 CpG sites show consistent hypomethylation in Symptomatic Parasitemia and a remarkable reversion of methylation state after malaria treatment (Figures 4A, B). We observed changes of TNF transcript abundance that are consistent with a model of local methylation regulation of TNF gene expression where hypomethylation in Symptomatic Parasitemia results in upregulation of TNF expression that then drops to pre-infection levels as the CpG site regain their methylation marks (Figure 4B). To test if methylation levels within each infection stage correlate with variation of TNF expression, we performed correlation analysis between the adjusted beta values of the 13 CpGs averaged and TNF expression levels in the three stages separately: Before Infection (*n* = 40), during Symptomatic Parasitemia infection (*n* = 40), and After Treatment (*n* = 40). The results show a significant negative correlation in the Symptomatic Parasitemia (*p*-value = 0.002) and After Treatment (*p*-value = 0.025) groups. In the Before Infection group, the trend was negative but not statistically significant (Figure 4C). Combined, these results support the hypothesis that the dynamic methylation changes taking place in the DMR result in changes in TNF gene expression levels that increase during symptomatic parasitemia and revert back to pre-infection levels. These results implicate methylation in the molecular mechanisms of TNF regulation in human malaria.

## 4 Discussion

Methylation of DNA plays a central role in gene regulation by controlling the accessibility of DNA to transcription factors and RNA polymerase. DNA methylation is a relatively stable epigenetic mark (Kim and Costello, 2017). However, under certain cellular environments (i.e., innate immune cells facing a parasitic infection), DNA methylation levels can change rapidly (Pacis et al., 2019). Although changes of DNA methylation in response to infection has received increased attention in the past years (Poulin and Thomas, 2008; Gómez-Díaz et al., 2012; Wenzel and Piertney, 2014; Deng et al., 2019; Dobbs et al., 2022; Bouyahya et al., 2022; Ding et al., 2022; Kaimala et al., 2022), little is known about the epigenetic response taking place in response to the malarial parasite *P. falciparum*. In this study, we generated the first matched genome-wide DNA methylation data at three time points in malaria. To our knowledge, this is the first report documenting host temporal DNA methylome changes in malaria.

Both supervised and unsupervised analysis of methylation data demonstrate significant changes of circulating immune cell methylation profiles. We document these major changes at DMP and DMR scales. During the symptomatic parasitemia stage, most of the DMPs and DMRs are hypomethylated. The relationship between DNA methylation and gene expression is complex but in general, hypomethylation is associated with increased transcription levels (Feinberg and Tycko, 2004; Nishigaki et al., 2005; Jones, 2012). Given the need for a rapid response of circulating immune cells to *Plasmodium* infection, the observed global hypomethylation response is expected and results in global transcriptional responses as previously reported in malaria (Idaghdour et al., 2012; Dieng et al., 2020). General hypomethylation was also reported to affect intergenic regions of DNA, including transposable elements, which can lead to changes in chromosomal stability (Wöhrle et al., 2001; Nichol and Pearson, 2002). Our results are consistent with other studies that have reported frequent occurrence of both global and localized DNA hypomethylation across a range of diseases including various types of cancers such as pancreas cancers (Sato et al., 2003), colon cancers (Frigola et al., 2005), breast cancer (Piotrowski et al., 2006), and neoplasia (Santini et al., 2001). Additionally, hypomethylation has been observed in other conditions such as Crohn’s disease (Somineni et al., 2019) and bacterial infection of human dendritic cells (Pacis et al., 2015). The methylation profile of circulating immune cells has undergone a significant reversal to the largely hypermethylated pre-infection state following treatment, with the majority of the DMPs and DMRs becoming hypermethylated. A study on pediatric patients with Crohn’s disease found similar DNA methylation patterns in blood samples, including changes in CpG methylation that disappeared with treatment of inflammation (Somineni et al., 2019). That said, a number of DMPs and DMRs are exceptions to this trend and suggest that *P. falciparum* infection and/or drug treatment leave an epigenetic footprint in the genomes of circulating immune cells. Regions that did not revert back may act as informed enhancers, potentially allowing for faster transcriptional responses to future infections. We speculate that the observed dynamic methylation changes in response to infection and treatment leave epigenetic signatures that could impact the host response to future infections.

The spatial density of DMPs and DMRs, accounting for the distribution of CpG sites on the EPIC array, revealed that chromosomes 1, 6, 11, 17, and 19 are the most subject to these changes. These chromosomes have the highest densities of immune genes indicating the occurrence of major epigenetic regulation of immune response. The majority of DMRs overlapped with gene bodies and promoters of master immune genes (i.e., HLA-DQB1, HLA-A, HHLA2, CD1C, IFNA1, IFNK, LY9, TNFSF18 and others). Notably and as anticipated, the highest number of DMRs implicating immune genes was observed during the symptomatic parasitemia phase, reflecting a systematic epigenetically-regulated transcriptional response to *P. falciparum* infection. Enrichment analysis of DMRs implicated several core immunological pathways that are crucial for the activation and regulation of immune response against malaria infection. The top impacted processes include immunoglobulin production, neutrophil degranulation, T cell activation and myeloid leukocyte differentiation, migration and chemotaxis. These processes play important roles in the pathogenesis of malaria (Sun and Chakrabarti, 1985; Tecchio and Cassatella, 2016; Aitken et al., 2018; Silveira et al., 2018) and potentially in the development of protective immunity (Uchechukwu et al., 2017). It is worth noting that the global methylation changes impacted non-immune system-related genes supporting the view that parasite infection affects various cellular processes beyond strict immune responses (Wenzel and Piertney, 2014).

Changes in DNA methylation profiles of *cis* and *trans* regulatory elements are predicted to result in changes of gene expression levels. Our TNF-focused integrative CpG methylation-TNF mRNA analysis implicates methylation in the molecular mechanisms of TNF regulation in human malaria. TNF is a vital component of the innate and adaptive immune systems and has been linked to the recruitment and enhancement of monocytes, neutrophils, and lymphocytes during inflammation triggered by malaria infection (Mackay et al., 1993; Pasparakis et al., 1996; Dobbs et al., 2022). Several previous studies have reported TNF *cis* regulatory allelic variation as well as altered levels of TNF expression associated with severe malaria infection (Henrici et al., 2021; Mahittikorn et al., 2022). Our integrative analysis identified the hypomethylated CpG sites that are most likely mediating the observed upregulation of TNF expression in response to *P. falciparum* expression. Hypermethylation of the same CpG sites is likely the mediator of downregulation of TNF expression observed following treatment. The matched nature of both our transcriptomic and methylation datasets enabled us to draw these inferences on the impact of DNA methylation changes on TNF gene expression.

In summary, we present the first genome-wide matched DNA methylation profiles of malarial children before infection, during symptomatic parasitemia and after treatment and provide evidence of widespread impact of *P. falciparum* on DNA methylation of circulating immune cells. Knowledge of epigenetic regulation of the human host to *P. falciparum* infection adds another layer of complexity to host-parasite interactions but offers also new insights into potential novel therapeutic approaches in malaria as has been done in other diseases.

## Data Availability

The 850k EPIC DNA methylation data is deposited in GEO under the following ID: GSE230850. URL: https://www.ncbi.nlm.nih.gov/geo/query/acc.cgi?acc=GSE230850.
