# Supplementary material for: Impact of Plasmodium falciparum infection on DNA methylation of circulating immune cells
Source: Front Genet. 2023 Jul 4;14:1197933. doi: 10.3389/fgene.2023.1197933 (PMC10352500; doi:10.3389/fgene.2023.1197933)
Supplement: Supplementary file 1 [file Table2.DOCX]

**Supplementary Information**


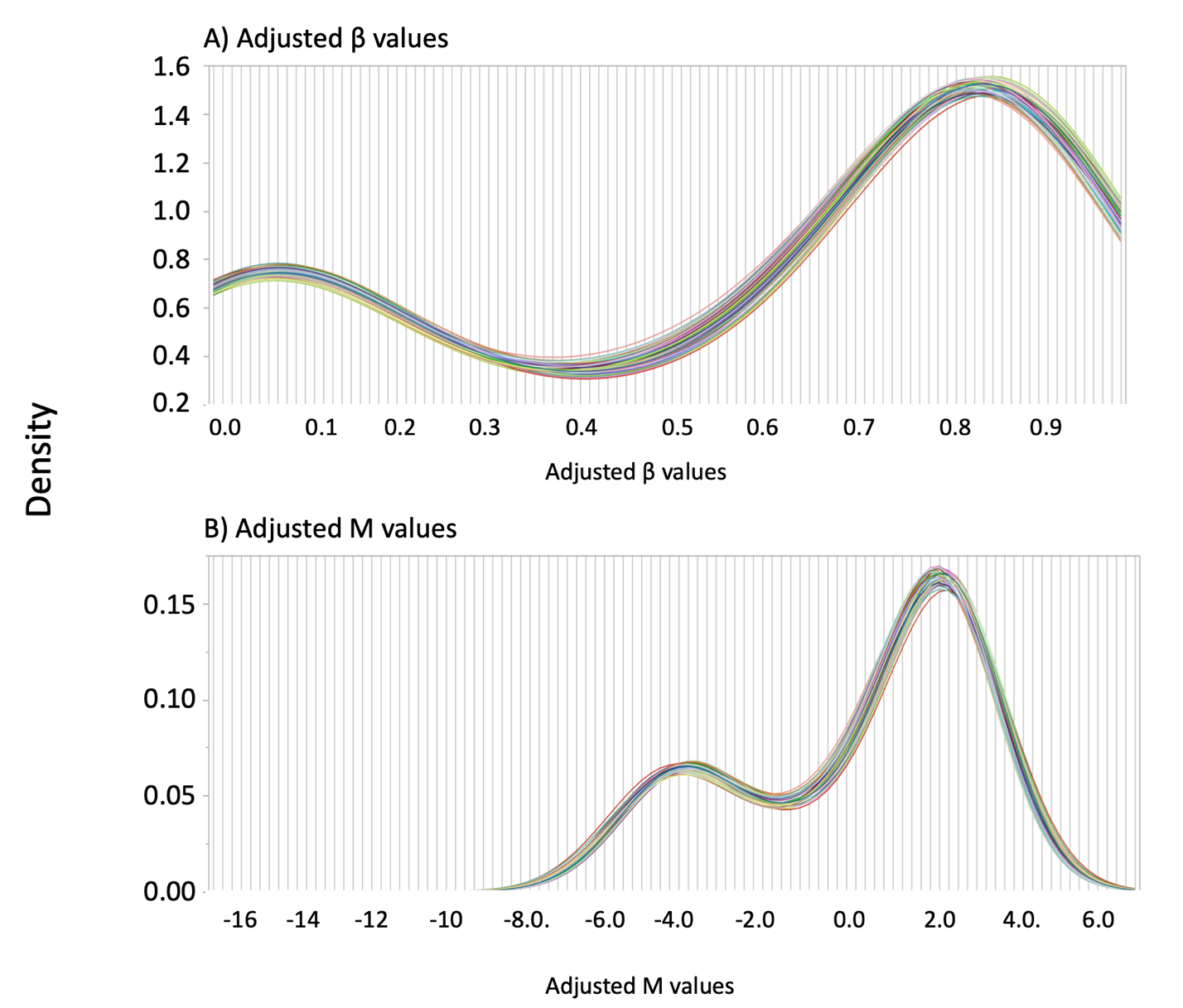


**Figure S1.** Density plots of normalized adjusted β values (A) and adjusted M values (B). Each line represents on individual study participant.

**
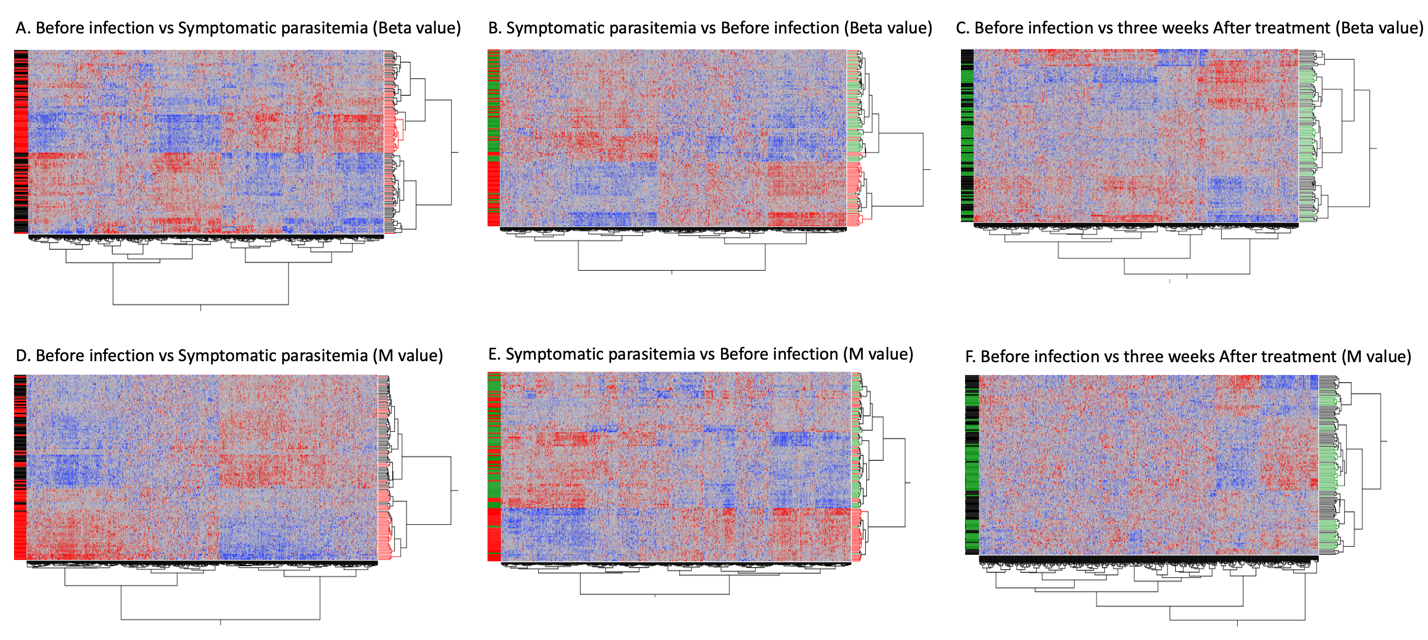
**

**Figure S2.** Heatmap of β values (A-C) and M values (D-F) of DMPs (columns) in each of the pairwise contrasts indicated. The β and M values in the heatmaps are colored from low to high (blue to gray to red). Each row represent one individual colored in the right and left side of the heatmap based on their infection status (Before infection, black; Symptomatic Parasitemia, red; After treatment, green.

**
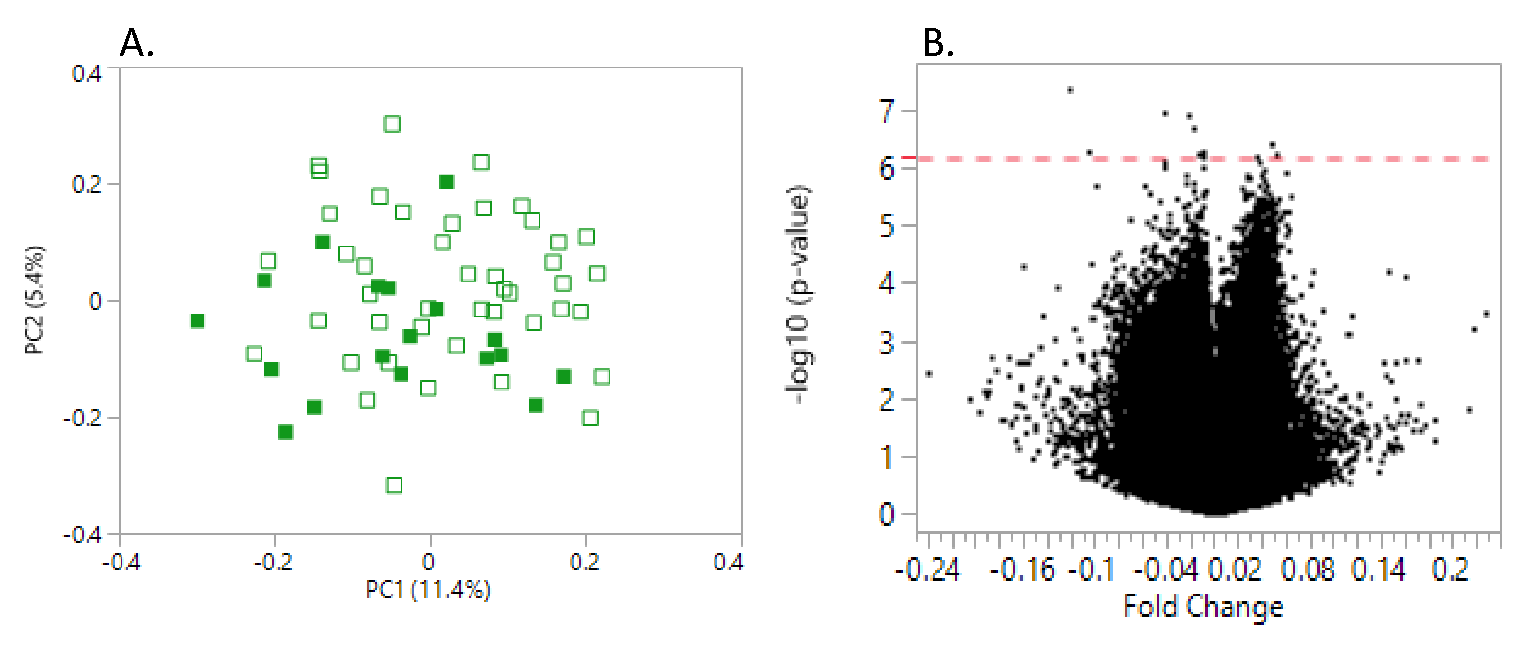
**

**Figure S3**. (A) PCA of DNA methylation profiles at the After Treatment (AT) stage. Children who cleared parasite three weeks after treatment are labeled as open squares and children who did not clear as solid squares. (B) Volcano plot of differential methylation (CpG = Age + Sex + Parasitemia clearance status). The horizontal red line indicates the 5% FDR significance threshold.

**
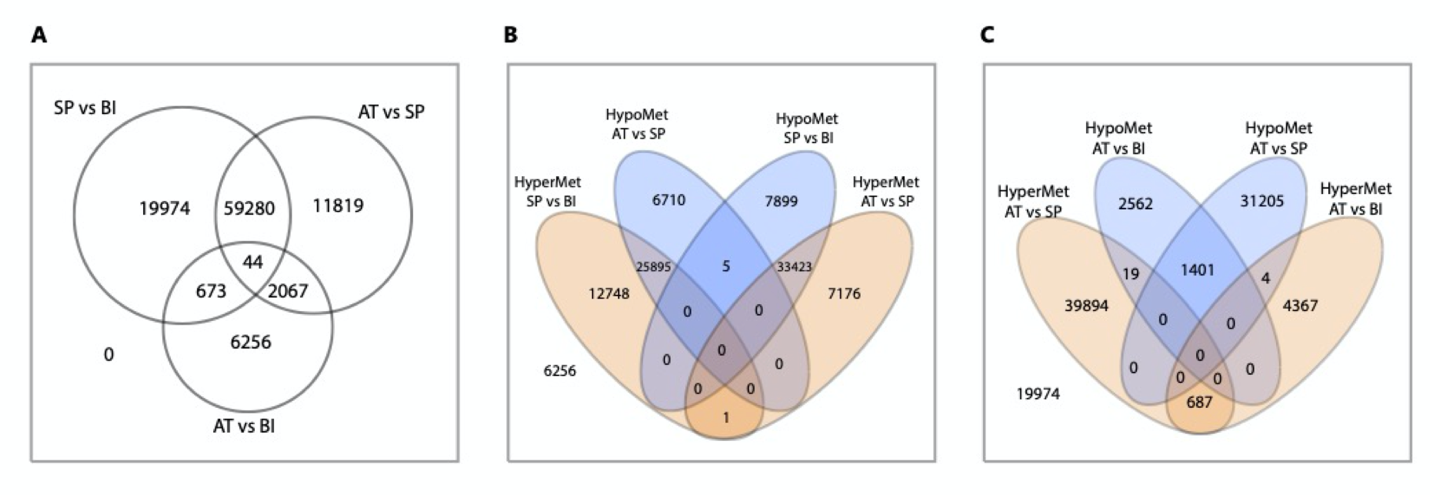
**

**Figure S4.** (A) Venn diagram of DMPs across three contrasts SP vs BI, AT vs SP and AT vs BI. (B) and (C) shows the breakdown of hypo- (blue) and hyper-methylated (orange) DMPs for the pairwise contrasts indicated.

**
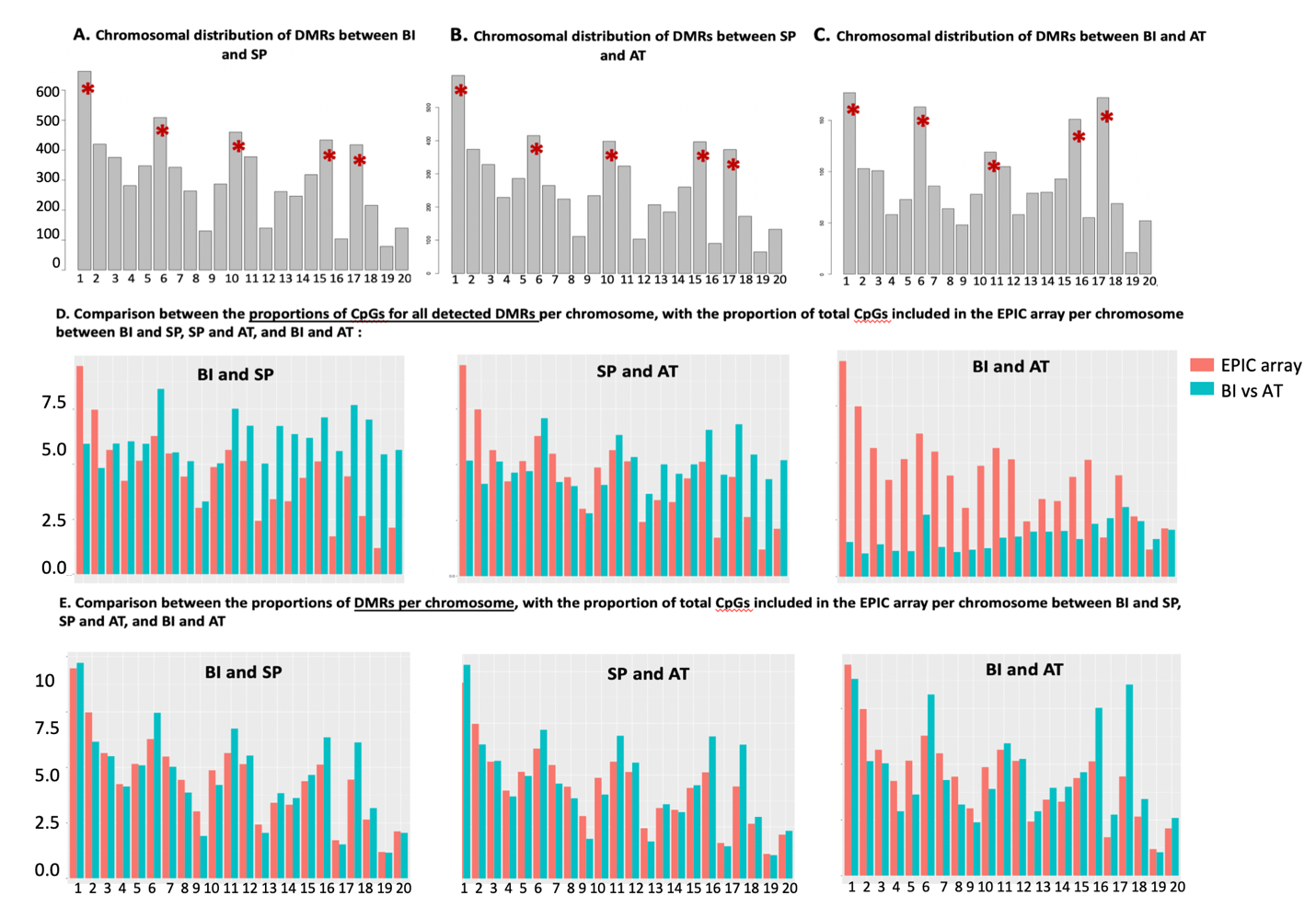
**

**Figure S5.** Distribution of differentially methylated regions (DMRs) along the chromosomes across the three stages investigated (A) Before infection (BI), (B) Symptomatic Parasitemia (SP) and (C) After Treatment (AT). (D) The proportion of CpGs for all detected DMRs per chromosome (blue bars) compared to the proportion of total CpGs in the EPIC array per chromosome (red bars) for each contrast: BI vs SP, SP vs AT and BI vs AT. (E) The proportion of DMRs per chromosome (blue bars) compared to the proportion of total CpGs in the EPIC array per chromosome (red bars) for each contrast: BI vs SP, SP vs AT and BI vs AT.

**
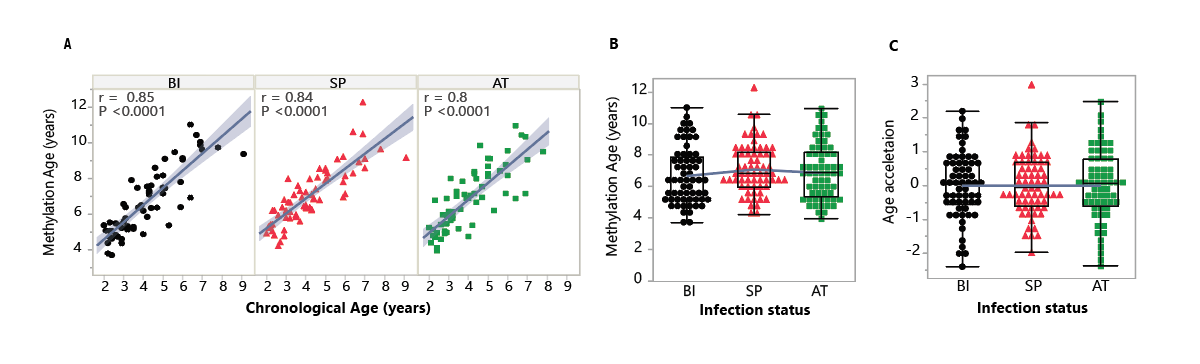
**

**Figure S6**. Methylation age changes across infection stages (Before infection (BI), black dots; Symptomatic parasitemia (SP), red triangles; After treatment (AT), green squares). (A) Scatter plots displaying the relationship between methylation and chronological age in children at each infection status: BI (r = 0.85, P < 0.0001), SP (r = 0.84, P < 0.0001), and AT (r = 0.80, P < 0.0001). (B) Changes in methylation age across the three stages: before infection (BI, black dots), symptomatic parasitemia (SP, red triangles), and after treatment (AT, green squares). Each point represents the DNA methylation age estimated using the Horvath et al. (2018) epigenetic clock model and methylation data from 391 CpGs. (C) The estimates of "age acceleration" for study participants in each of three stages.

**Supplementary Tables**

**Table S1. Biological parameters of the enrolled participants across the three stages sampled**. BI = Before Infection (n = 61), SP = Symptomatic Parasitemia (n = 66), and AT = After Treatment (n=62). Data are listed as median (minimum value – maximum value). The mean age of participants is 4 years (2 – 9.1).

|  | **BI** | **SP** | **AT** |
| --- | --- | --- | --- |
| **Lymphocytes** | 51.6 (24.1-71.2) | 27.9 (8.6-67.3) | 49.4 (21.7-72.5) |
| **Monocytes** | 7.1 (0.42-11.7) | 11 (4-19.8) | 8.1 (4.4-21.2) |
| **Neutrophils** | 37.3 (14.2-67.1) | 57.2 (21.2-82.5) | 36.9 (19.1-60.7) |
| **Eosinophils** | 3.2 (0.6-18.5) | 0.9 (0-9.3) | 2.9 (0.2-19.6) |
| **Basophils** | 0.7 (0.2-2.6) | 0.6 (0-4.7) | 0.6 (0.2-5.6) |
| **Parasitemia (log2)** | 0 | 15.3 (0-18.1) | 0 (0-17) |

**Table S2.** Summary information for the differentially methylated regions (DMRs) between three stages investigated.

|  | **No. CpG within a DMR** | **DMR width** | **Hyper** | **Hypo** |
| --- | --- | --- | --- | --- |
| **BI / SP** | 6 (5 – 56) | 497 (14 - 3588) | 1,184 | 5,634 |
| **SP / AT** | 6 (5 – 52) | 453 (23 - 2920) | 4,698 | 1,069 |
| **BI / AT** | 6 (5 – 47) | 207 (14 - 1519) | 808 | 1,197 |
